# The impact of weather and storm water management ponds on the transmission of West Nile virus

**DOI:** 10.1098/rsos.170017

**Published:** 2017-08-16

**Authors:** Yiyuan Wang, Wendy Pons, Jessica Fang, Huaiping Zhu

**Affiliations:** 1 LAMPS, Department of Mathematics and Statistics, York University, Toronto, Ontario, Canada; 2 Environmental Health, Peel Public Health, Ontario, Canada; 3 Toronto and Region Conservation Authority, Brampton, Ontario, Canada

**Keywords:** West Nile virus, temperature, precipitation, storm water management ponds, intraspecific competition

## Abstract

West Nile virus (WNV) is the most widely distributed arbovirus in the world and the spread is influenced by complex factors including weather conditions and urban environmental settings like storm water management ponds (SWMP). The purpose of this work was to develop an ordinary differential equation model to explore the impacts of SWMP, temperature and precipitation on WNV vector abundance and the transmission of WNV between mosquito and bird populations. The model was used to analyse how weather conditions and SWMP can influence the basic reproduction number. The results found that an excess of precipitation and fiercer intraspecific competition will reduce vector population and the peak value of infectious vectors and birds. This information can be used to identify measures that would be useful to control larval abundance in SWMP and the transmission of WNV.

## 1. Introduction

**Figure 1.**
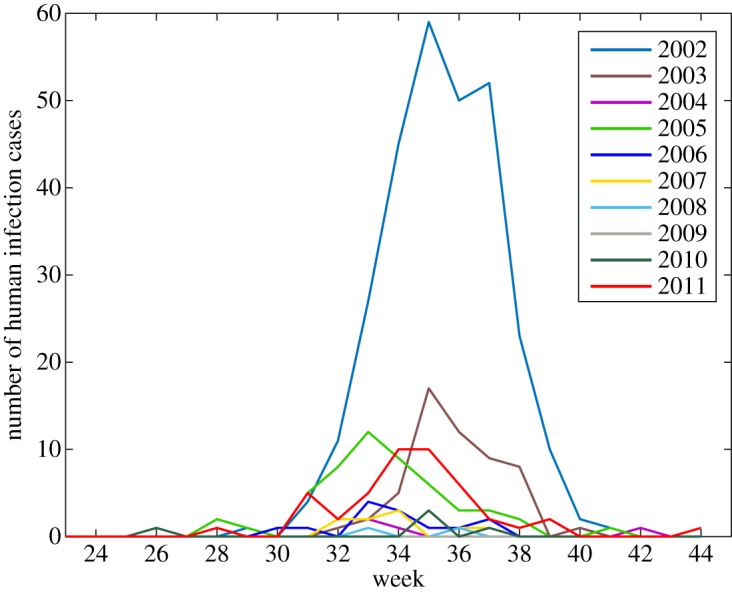
Human infections in the Greater Toronto Area from June to October, 2002–2011 (data from Public Health Ontario).

West Nile virus (WNV) is primarily a bird pathogen and a mosquito-borne arbovirus belonging to the genus *Flavivirus*. The female mosquito gets infected by feeding on the blood of birds carrying the virus and then transmits the virus to humans and other animals; the mammals are dead-end hosts [1–3]. WNV is the most widely distributed emerging arbovirus, with no specific treatment or vaccine for humans [4]. In North America, the first WNV case was detected in New York City in 1999; the virus spread rapidly throughout the continent and appeared in Ontario in 2001 [5,6]. Since 2001, human infections have occurred yearly in Ontario; the number of cases varies based on the time at which WNV becomes endemic and the peak value of infections (figure 1). The variations of the annual human infection may be due to a number of complex factors including vector–virus–host interactions, the increase in urbanization and agriculture, climatic factors, and anthropogenic land use such as the storm water management ponds (SWMP) [7–12].

SWMP, including wet ponds and dry ponds, are artificial ponds designed to collect, retain and filter storm water run-off [13]. In Ontario, municipalities first began implementing wet ponds as part of their storm water infrastructure in the late 1980s. Currently, there are over 1000 SWMP and wetlands in the Greater Toronto Area (GTA) [14]. Improperly designed, operated and maintained SWMP can be conducive to creating standing water. Particularly for wet ponds that maintain a permanent pool of water, shallow zones of these ponds may be an attractive fertile breeding site for the female *Culex* mosquitoes. Thus the SWMP, along with temperature, precipitation and wind patterns, can contribute to supporting the growth and development of mosquitoes that are competent WNV vectors.

The Toronto and Region Conservation Authority (TRCA) has been running a WNV mosquito larval monitoring and surveillance programme in natural wetlands and SWMP on TRCA lands in the GTA since 2003. Their results showed that the mosquitoes collected from these SWMP were principally WNV vector species, predominantly *Culex pipiens*. SWMP can be used to predict adult mosquito emergence and the potential for human infections [6]. Research also indicates that larval abundance is related to temperature and also to precipitation [15].

Mathematical modelling for mosquito abundance and the transmission of WNV has been studied extensively [16–22]. An ordinary differential equation model in [23] showed that mosquito control can prevent a WNV outbreak. To simulate the population dynamics of immature and adult *Culex* mosquitoes in the Northeastern USA, Gong *et al.* [24] developed climate-based models and revealed a strong correlation between the timing of early population increases and decreases in late summer. Additionally, the influence of weather conditions on the mosquito population or infection were studied in [25,26].

Previous mathematical modelling has failed to take into account the impact of SWMP in a dynamical model. The purpose of this research was to develop a single-season dynamical model between mosquito and bird populations to explore the influences of SWMP and weather conditions on vector abundance and the transmission of WNV.

The better understanding of the mechanism of a WNV outbreak and having a more reliable evaluation of transmission risk will greatly help to control the spread of the virus and human infections. In our work, we will build a WNV transmission model among mosquitoes and birds. We will split the mosquito population in two stages; furthermore, we consider the intraspecific competition of mosquitoes in the aquatic stage. We will yield new insights into the transmission of WNV and the threshold conditions of a WNV outbreak. Moreover, we will propose a novel index to assess the risk of WNV transmission.

## 2. Methods

To model the SWMP influence on the mosquito population, we created a two-stage mosquito population model to investigate the role of intraspecific competition on the development and abundance of *Culex* mosquitoes. We applied the intraspecific competition rate in aquatic stages of mosquitoes: the abundance of pre-adults is closely related to intraspecific competition, and intraspecific competition is associated with standing water developed from the water in SWMP.

A second model was developed to explore the impact of SWMP on the transmission of WNV with or without the weather factors. We combined the host birds and the vector mosquitoes and established a single-season compartmental model. To incorporate weather factors, we first identified critical input parameters of this model by performing sensitivity analysis. Then we extended it into a weather-driven model, where SWMP in conjunction with precipitation determines the water habitat for larvae, with the weather data from June to October gathered from Toronto Pearson International Airport Station [27].

### 2.1. Two-stage *Culex* mosquito population model

Intraspecific competition is an interaction in population ecology. Members of the same species compete for limited resources required for survival and development [28]. Competition among larvae is an influential factor regulating the growth of mosquito populations [29]. For instance, *C. pipiens* experience density-dependent reductions in growth and survival at the larval stage [29–32].

The logistic growth equation is used to model intraspecific competition in biological systems. It depicts the reciprocal relation between the carrying capacity and the intraspecific competition rate [33]. The major impact of intraspecific competition is reduced population growth rates as population density increases. The intraspecific competition rate is assumed to be relevant to any element of competition like the size of standing water and the density of nutrients. With these assumptions, our mosquito model is given by

2.1 $$\begin{array}{rl} \frac{dL}{dt} & =rM-\delta L-d_{l}L-\kappa L^{2}\\ \;\text{and}\;\;\frac{dM}{dt} & =\delta L-d_{m}M, \end{array}\}$$

where *L*(*t*) is the population of pre-adult mosquitoes (eggs, larvae, pupae) at time *t*, *M*(*t*) is the population of adult mosquitoes at time *t*, *r* is the *per capita* birth rate of mosquitoes, *δ* is the *per capita* maturation rate of mosquitoes from the pre-adult stages, *κ* is the intraspecific competition rate and *d*_l_ and *d*_ml_ are the *per capita* mortality rates of pre-adult and adult mosquitoes, respectively.

### 2.2. Mosquito–bird model without weather factors

Owing to WNV circulating between mosquitoes and birds and being established as a seasonal epidemic in North America, we extended the mosquito–bird model in [23] and developed a single-season ordinary differential equation model on WNV transmission in the mosquito–bird population. For the mosquito population, we adopt the two-stage *Culex* mosquito model (2.1), assuming pre-adult mosquitoes include both female and male with the sex ratio 1:1 [34–36] and competitive interactions are within and between both female and male [29]. For intraspecific competition, we assume it is only related to the size of standing water; other factors such as density of nutrients and oxygen are fixed. Adult female mosquitoes are classified into susceptible, exposed and infectious compartments. For avian hosts, more than 300 species of birds are involved in the WNV transmission in North America [37]. Here, focusing on the effects of SWMP and for simplicity, we regard all birds as one family and classified the family into susceptible, infectious, recovered and dead compartments. In this single-season (from spring to autumn) model, it is reasonable that the demographic dynamics of mosquitoes is considered but not for birds. We further make assumptions that vertical transmission in mosquitoes and horizontal transmission in birds are small and neglected [23]. For an accurate estimation of the WNV epidemic, we consider mammals which are dead-end hosts also providing blood meals for mosquitoes [17]. Then, our model (all parameters are defined in table 1) is

2.2 $$\begin{array}{rl} \frac{dL_{m}}{dt} & =r_{m}(S_{m}+E_{m}+I_{m})-\delta L_{m}-d_{l}L_{m}-\kappa L_{m}^{2},\\ \frac{dM_{m}}{dt} & =\frac{1}{2}\delta L_{m}-{\tilde{d}}_{m}M_{m},\\ \frac{dS_{m}}{dt} & =\frac{1}{2}\delta L_{m}-b_{m}\beta_{m}S_{m}\frac{I_{b}}{N_{b}+A}-d_{m}S_{m},\\ \frac{dE_{m}}{dt} & =b_{m}\beta_{m}S_{m}\frac{I_{b}}{N_{b}+A}-kE_{m}-d_{m}E_{m},\\ \frac{dI_{m}}{dt} & =kE_{m}-d_{m}I_{m},\\ \frac{dS_{b}}{dt} & =-b_{m}\beta_{b}I_{m}\frac{S_{b}}{N_{b}+A},\\ \frac{dI_{b}}{dt} & =b_{m}\beta_{b}I_{m}\frac{S_{b}}{N_{b}+A}-\mu I_{b}-\gamma I_{b},\\ \frac{dR_{b}}{dt} & =\gamma I_{b}\\ \;\text{and}\;\;\frac{dX_{b}}{dt} & =\mu I_{b}, \end{array}\}$$

where *L*_ml_(*t*) is the population of pre-adult WNV vector mosquitoes at time *t*, *M*_ml_(*t*) is the population of male adults developed from the pre-adult stages at time *t*; *S*_ml_(*t*),*E*_ml_(*t*) and *I*_ml_(*t*) are the populations of susceptible, exposed and infectious female mosquitoes, respectively, at time *t*; *S*_b_(*t*),*I*_b_(*t*),*R*_b_(*t*) and *X*_b_(*t*) are the populations of susceptible, infectious, recovered and dead birds, respectively, at time *t*; *A* is the total number of mammals that mosquitoes feed on for blood meals and *N*_b_=*S*_b_+*I*_b_+*R*_b_.

**Table 1.** Parameters in WNV model.

parameters	interpretation	baseline(range)
*r*_ml_	mosquitoes *per capita* birth rate (or oviposition rate)	0.6(0.036,42.5) [23]
*δ*	mosquitoes *per capita* maturation rate from pre-adult stages to adult	0.06(0.051,0.093) [23]
*d*_l_	pre-adult mosquitoes *per capita* mortality rate	0.4(0.213,16.9) [23]
*κ*	intraspecific competition rate of pre-adult mosquitoes	0.005(0,1)
*b*_ml_	female adult mosquitoes *per capita* biting rate	0.5(0.2,0.75) [17]
*β*_ml_	WNV transmission probability from birds to mosquitoes	0.12(0.02,0.24) [23]
*d*_ml_	female adult mosquitoes *per capita* mortality rate	0.05(0.016,0.07) [23]
${\tilde{d\;}\;}_{m}$	male adult mosquitoes *per capita* mortality rate	0.05(0.016,0.07) [23]
*k*	female adult mosquitoes *per capita* transition rate from exposed to infected	0.09(0.087,0.125) [23]
*β*_b_	WNV transmission probability from mosquitoes to birds	0.84(0.8,1.0) [23]
*μ*	birds *per capita* mortality rate due to WNV	0.127(0.125,0.2) [23]
*γ*	birds *per capita* recovery rate from WNV	0.001(0,0.2) [17]

### 2.3. Mosquito–bird model incorporating temperature and precipitation

Environmental factors, especially temperature and precipitation, largely impact the transmission of mosquito-borne pathogens by affecting the infection rate of the virus as well as mosquito and host abundance in time and space [38]. We explore a WNV transmission model between mosquitoes and birds driven by major environmental factors including temperature and precipitation.

#### 2.3.1. Sensitivity analysis

To incorporate temperature and precipitation, we first identify which input parameters significantly contribute to model outcomes. The identification is implemented by studying the sensitivity analysis of each parameter in system (2.2) on the two critical output variables, the population of adult mosquitoes *N*_m_ (*N*_m_=*M*_m_+*S*_m_+*E*_m_+*I*_m_) and the basic reproduction number *R*_0_ (3.1). Specifically, we evaluate partial rank correlation coefficients (PRCCs) between each input parameter and output variable, using Latin hypercube sampling (LHS) with 3000 samples [39]. Owing to the absence of available data and *a priori* information on the distributions of input parameters, we choose uniform distributions for each parameter with corresponding baseline and range in table 1. Obtained (from §3), the parameters with the most impact on both *N*_m_ and *R*_0_ are adult mosquito biting rate, oviposition rate and mortality rate, and pre-adult mosquito maturation rate, mortality rate and intraspecific competition; we incorporate temperature and precipitation into these six critical input parameters.

#### 2.3.2. Temperature-dependent parameters

Temperature can affect mosquito development, infection and dissemination of WNV; it is especially influential in *Culex* mosquito biting activity, oviposition rate, maturation and mortality rates [24,36,40–43]. Mosquito biting rate and oviposition rate were fitted to the reciprocal of the duration of the mosquito gonotrophic cycle; maturation rate is described by the Sharpe & DeMichele equation; and mortality rates are U-shaped functions with respect to temperature [24,44,45]; specifically, these temperature-dependent rates were (related parameters are defined in table 2)

2.3 $$\begin{array}{rl} b_{m}(T) & =\frac{0.344}{1+1.231\exp(-0.184(T-20))}, \end{array}$$

2.4 $$\begin{array}{rl} r_{m}(T) & =cb_{m}(T), \end{array}$$

2.5 $$\begin{array}{rl} \delta (T) & =\frac{AA(T+273.15)}{298.15}\frac{\exp[(HA/1.987)(1/298.15-1/(T+273.15))]}{1+\exp[(HH/1.987)(1/TH-1/(T+273.15))]}, \end{array}$$

2.6 $$\begin{array}{rl} d_{l}(T) & =1-S_{l}\exp\left[-{\left(\frac{T-T_{l}}{{\mathrm{Var}}_{Tl}}\right)}^{2}\right] \end{array}$$

2.7 $$\begin{array}{rl} \;\text{and}\;d_{m}(T) & =1-S_{m}\exp\left[-{\left(\frac{T-T_{m}}{{\mathrm{Var}}_{Tm}}\right)}^{2}\right]. \end{array}$$

**Table 2.** Temperature-dependent and precipitation-dependent parameters in model (2.3.4).

parameters	interpretation	range
*c*	the scaling factor associated with biting rate	2.325 [44]
*AA*	parameters related to the individual	0.25 [24]
*HA*	thermodynamic characteristics	28 094 [24]
*HH*	of the organism’s control	35 362 [24]
*TH*	enzyme system	298.6 [24]
*T*_l_	optimal temperature for survival of pre-adult mosquitoes^a^	17 [24]
*T*_m_	optimal temperature for survival of female adult mosquitoes^a^	23 [24]
$T_{\tilde{m}}$	optimal temperature for survival of male adult mosquitoes^a^	23 [24]
*S*_l_	survival rates of pre-adult mosquitoes with *T*_l_	0.6–0.95 [24]
*S*_m_	survival rates of female adult mosquitoes with *T*_m_	0.6–0.95 [24]
$S_{\tilde{m}}$	survival rates of male adult mosquitoes with $T_{\tilde{m}}$	0.6–0.95 [24]
Var_*Tl*_	variance of *T*(*t*)^b^	
Var_*Tm*_	variance of *T*(*t*)^b^	
${\mathrm{Var}}_{T\tilde{m}}$	variance of *T*(*t*)^b^	
*P*_l_	optimal amount of precipitation	5 [27]
$\bar{\kappa }$	intraspecific competition rate when *P*=*P*_l_	0–1^*c*^
*ρ*>0	the scaling factor to reflect the amplitude of the *κ*^d^	
Var_*Pl*_	variance of *P*(*t*)^b,d^	

^a^All temperature parameters are in degree Celsius [24,44,46,47].

^b^Calculated with temperature and precipitation data in the GTA.

^c^Derived from the reciprocal of carrying capacity ranging from 1 to any positive integer.

^d^$(1+\rho )\bar{\kappa }$ represents the maximum value of *κ* with the constraint $0<(1+\rho )\bar{\kappa }<1$.

#### 2.3.3. Precipitation-dependent parameter

Precipitation influences the mosquito life cycle in two principal aspects: (i) the increased near-surface humidity related with precipitation promotes mosquito flight activity and host-seeking behaviour and (ii) precipitation can change the abundance and type of aquatic habitats where mosquitoes oviposit and the subsequent development of the aquatic stages [48]. In our study, we primarily take into account the second influence associated with the SWMP; in particular, precipitation in conjunction with SWMP has a profound effect on the intraspecific competition rate *κ* [49].

Based upon larvae data from TRCA, weather data in the GTA [27] and previous work [15], we determined that moderate precipitation is needed to provide habitats for *Culex* larvae depositing eggs and development. Nevertheless, an excess of precipitation will dilute or even refresh standing water, increasing the intraspecific competition. In this situation, the approximate description of the carrying capacity function relative to precipitation resembles the Gaussian function. By $\bar{K}$, we denote the carrying capacity with optimal precipitation and we adopt the relation

2.8 $$K(P)=\frac{\bar{K}}{1+\rho }\left(1+\rho \exp\left[-{\left(\frac{P-P_{l}}{{\mathrm{Var}}_{Pl}}\right)}^{2}\right]\right),$$

By combining the reciprocal relationship between carrying capacity and intraspecific competition, we propose a precipitation-dependent intraspecific competition rate as:

2.9 $$\kappa (P)=\frac{(1+\rho )\bar{\kappa }}{1+\rho \exp[-{\left((P-P_{l})/{\mathrm{Var}}_{Pl}\right)}^{2}]},$$

here $\bar{\kappa }=1/\bar{K}$ and all parameters are defined in table 2. Particularly, *ρ* and $\bar{\kappa }$ are largely dependent on the properties of the SWMP itself, like the size and depth of the pond.

#### 2.3.4. Formulation of the model

To formulate the transmission model with weather factors (denoted as model (2.3.4)), we assume that the mean daily temperature of breeding sites is equal to the mean daily temperature of the air because of a lack of water temperature data. Replacing parameters *b*_m_, *r*_m_, *δ*, *d*_l_, *d*_m_ and *κ* in (2.2) by *b*_m_(*T*) (2.3), *r*_m_(*T*) (2.4), *δ*(*T*) (2.5), *d*_l_(*T*) (2.6), *d*_m_(*T*) (2.7) and *κ*(*P*) (2.9), respectively, we have ${\tilde{d}}_{m}(T)=1-S_{\tilde{m}}\exp[-{\left((T-T_{\tilde{m}})/{\mathrm{Var}}_{T\tilde{m}}\right)}^{2}]$.

For the model with the daily changing temperature and precipitation, we replace the fixed temperature *T* and precipitation *P* in (2.3.4) by *T*(*t*) and *P*(*t*), and simulate the transmission dynamics based on weather data (see appendix C). Here, the maturation rate is treated specially because it relies on the average temperature of several days, while other weather-dependent parameters only depend on the temperature or precipitation of a single day. More specifically, the maturation rate on the *n*th day is influenced by the arithmetic means of the daily mean temperatures of 11 days before the *n*th day [25], i.e. $\frac{1}{11}\sum_{i=n-1}^{n-11}T_{i}$; other parameters are only related to *T*_*n*_ or *P*_*n*_, where *T*_*i*_ and *P*_*i*_ are the daily mean temperature and daily total precipitation on the *i*th day, respectively. To determine the influence of precipitation, we select three distinguished patterns of precipitation: normal precipitation, heavy precipitation (30 mm more daily) and heavier precipitation (60 mm more daily). The three different patterns are applied to a single month—July or September, as the mosquito population, infectious mosquitoes and birds increase in July and decrease in September.

## 3. Results

Sensitivity analysis indicates that *Culex* mosquito biting rate, oviposition rate, maturation rate, mortality rates and intraspecific competition rate significantly influence both mosquito abundance *N*_m_ and an indicator of local WNV activity level *R*_0_; and the effects of weather factors reflected by these rates. Mathematical model (2.3.4) shows that moderate temperature and precipitation, as well as a smaller intraspecific competition rate are more likely to result in the basic reproduction number greater than one, resulting in an outbreak of WNV.

When weather conditions are varied along with time, applying the daily temperature and precipitation in the GTA into our model, the numerical simulations display that a smaller intraspecific competition rate related to SWMP will lead to a larger total population of mosquitoes, infectious vectors and hosts. Additionally, total vector abundance and the peak value of infectious vectors and birds will be restricted by excessive precipitation.

### 3.1. Models analysis

For the two-stage *Culex* mosquito population model, it is evident that the intraspecific competition rate *κ* restrains the growth and development of the mosquitoes; when the intraspecific competition is fierce (*κ* is quite large), the immature mosquito population will decrease, resulting in the reduction of total number of mosquitoes. For the transmission models, the intraspecific competition has a similar effect.

Model (2.2), without weather factors, has up to two disease-free equilibrium (DFE) points. If *r*_m_*δ*/2*d*_m_−(*d*_l_+*δ*)<0, the model has a unique locally stable equilibrium point *E*_0_. If *r*_m_*δ*/2*d*_m_−(*d*_l_+*δ*)>0, the model has two DFE points, an unstable *E*_0_, and *E*_1_ whose local stability is determined by the basic reproduction number *R*_0_ obtained from the next-generation matrix of the system (2.2):

3.1 $$R_{0}=\sqrt{\frac{b_{m}\beta_{m}(S_{m_{0}}/(N_{b_{0}}+A))(k/(k+d_{m}))}{\left(\mu +\gamma \right)}\frac{b_{m}\beta_{b}(S_{b_{0}}/(N_{b_{0}}+A))}{d_{m}}}.$$

From a biological perspective, *R*_0_ gives the expected number of new infections produced by a single infective mosquito or bird when introduced into a susceptible population. When *R*_0_<1, *E*_1_ is locally stable; when *R*_0_>1, *E*_1_ is unstable [50].

We find that *κ*, reflecting the role of SWMP, only affects the stability of *E*_1_. Other aspects related to SWMP, such as the surroundings and the size of a pond, still only influence *κ* and the stability of *E*_1_ accordingly. Furthermore, the threshold level of the intraspecific competition related to SWMP derived from the threshold *R*_0_=1 is

3.2 $$\kappa =\frac{b_{m}^{2}S_{b_{0}}\beta_{m}k\beta_{b}\delta [r_{m}\delta -2d_{m}(d_{l}+\delta )]}{4{\left(N_{b_{0}}+A\right)}^{2}d_{m}^{3}(\mu +\gamma )(k+d_{m})}≐\kappa^{*}.$$

If the intraspecific competition is not strong (*κ*>κ*), for instance, a pond is located among plants where fertilizers are applied, this pond receiving many nutrients can favour submersed aquatic vegetation and algae blooms, which create more ideal habitats for larvae and hence weaken the intraspecific competition; then *E*_1_ is unstable and the disease introduction will lead to an outbreak. Otherwise, strong competition (*κ*>κ*) results in controlling mosquito abundance and even preventing a WNV outbreak.

Fixing temperature and precipitation, the basic reproduction number can still be obtained as

3.3 $${\hat{R}}_{0}=\sqrt{\frac{k\beta_{m}\beta_{b}S_{b_{0}}b_{m}{\left(T\right)}^{2}\delta (T)[r_{m}(T)\delta (T)/2d_{m}(T)-(d_{l}(T)+\delta (T))]}{2{\left(N_{b_{0}}+A\right)}^{2}(\mu +\gamma )d_{m}{\left(T\right)}^{2}(k+d_{m}(T))\kappa (P)}}.$$

We see that ${\hat{R}}_{0}$ initially increases and then decreases as precipitation *P* increases, and reaches the maximum value at *P*_l_. The closer the amount of precipitation gets to the optimal amount of precipitation, the higher is the probability of occurrence of a WNV outbreak. Also, a larger $\bar{\kappa }$ or *ρ* (when *P*≠*P*_l_) could prevent a WNV outbreak from occurring because an increase of $\bar{\kappa }$ or *ρ* leads to a decrease of ${\hat{R}}_{0}$.

### 3.2. Numerical simulations

Numerical simulations display the influence of each parameter of system (2.2) on the outcome variable *N*_m_ and *R*_0_, the impact of precipitation and SWMP on the transmission of WNV and the vector population, where both fixed weather conditions and time-dependent weather conditions are included.

**Figure 2.**
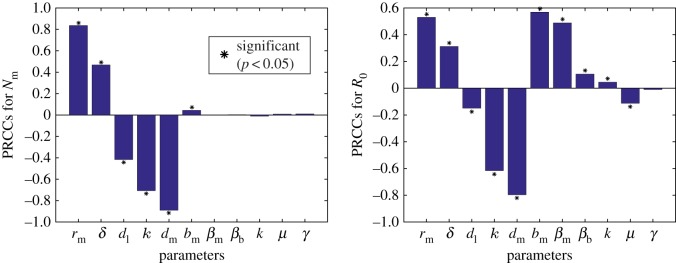
Performance of LHS/PRCC on the model (2.2). Parameters with a PRCC significantly (*p*<0.05) different from zero are indicated with (*). (*a*) PRCCs between input parameters and the outcome variable *N*_m_ and (*b*) PRCCs between input parameters and the outcome variable *R*_0_.

Sensitivity analysis (figure 2) shows that mosquito oviposition rate *r*_m_, maturation rate *δ*, mortality rates *d*_l_ and *d*_m_, intraspecific competition rate *κ* and biting rate *b*_m_ have significant impacts (with *p*<0.05) on both mosquito population *N*_m_ and indicator of local WNV severity *R*_0_. For other parameters, *R*_0_ is also sensitive to WNV transmission probability *β*_m_ and *β*_b_, bird transition rate from the exposed to the infected *k* and WNV-induced mortality rate *μ*, while these parameters have only slight impacts on *N*_m_. Decreasing *r*_m_, *δ* and *b*_m_ or increasing *d*_l_, *κ* and *d*_m_ can lead to the simultaneous decrease in *N*_m_ and *R*_0_, which is beneficial to control mosquitoes and WNV transmission.

**Figure 3.**
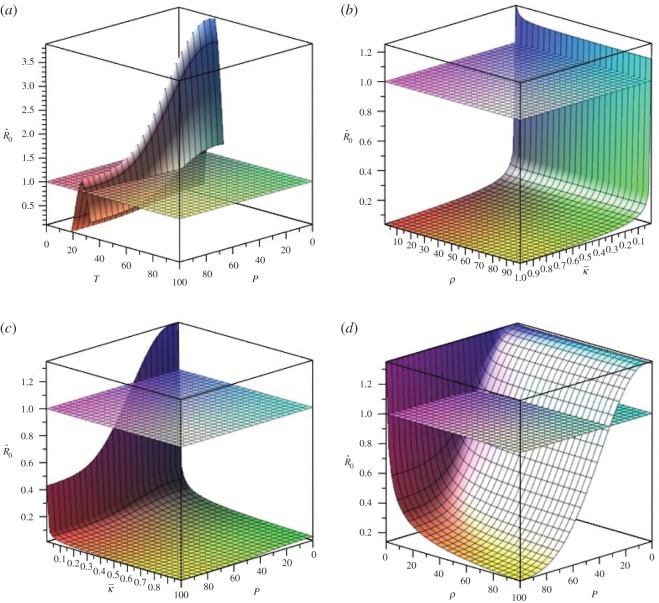
Variation of basic reproduction number ${\hat{R}}_{0}$ along with combinations of weather conditions or SWMP properties. (*a*) Weather conditions, (*b*) SWMP properties, (*c*) precipitation with $\bar{\kappa }$ of SWMP and (*d*) precipitation with *ρ* of SWMP.

Incorporating weather factors in these critical parameters, when weather conditions do not change with time (figure 3), too high or too low temperature or a heavier precipitation will decrease the basic reproduction number ${\hat{R}}_{0}$, leading to a lower risk of WNV. For SWMP, a larger intraspecific competition rate $\bar{\kappa }$ and a larger scaling factor *ρ* can make the ${\hat{R}}_{0}$ less than one, controlling the vector abundance and the WNV transmission. In figure 3*b*,*c*, ${\hat{R}}_{0}>1$ is for a small part of parameters space, and ${\hat{R}}_{0}$ increases rapidly in these fringe conditions. That is, when the intraspecific competition among pre-adult mosquitoes is weak, for instance with less restriction of habitats (i.e. with sufficient standing water in the pond), more mosquitoes will be developed and involved in WNV transmission, leading to a high potential of WNV outbreak. Particularly, when the intraspecific competition barely exists, reproduction of mosquitoes will increase dramatically.

**Figure 4.**
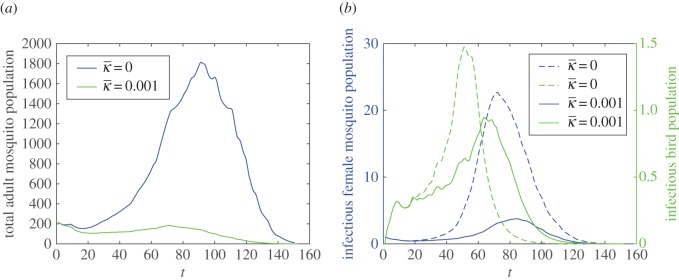
The impact of $\bar{\kappa }$ on the mosquito abundance and transmission of WNV. (*a*) The impact of $\bar{\kappa }$ on the total number of adult mosquitoes and (*b*) the impact of $\bar{\kappa }$ on infectious female mosquitoes and birds.

**Figure 5.**
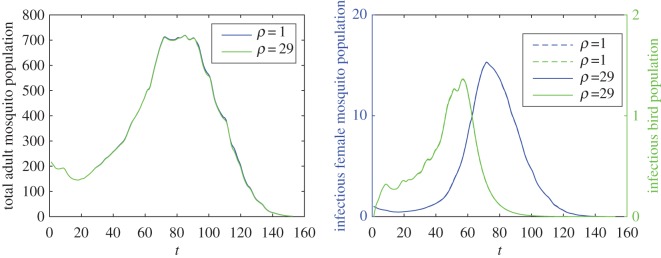
The impact of *ρ* on the mosquito abundance and the transmission of WNV. (*a*) The impact of *ρ* on total adult mosquitoes and (*b*) the impact of *ρ* on infectious female mosquitoes and birds.

**Figure 6.**
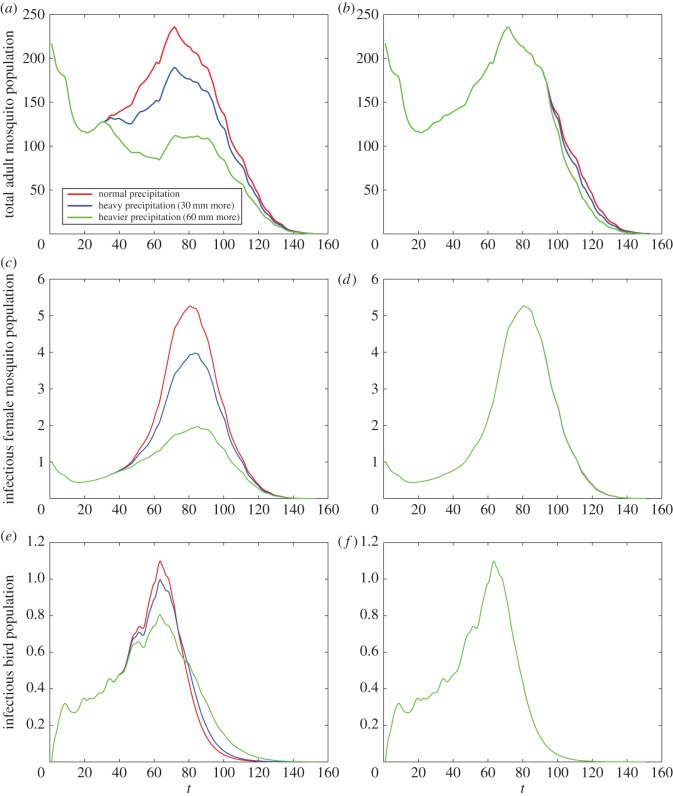
The impact of precipitation on mosquito abundance and the transmission of WNV with $\bar{\kappa }=0.0007$ and *ρ*=9. (*a*) Total adult mosquito population based on different patterns of precipitation in July, (*b*) total adult mosquito population based on different patterns of precipitation in September, (*c*) infectious female mosquito population based on different patterns of precipitation in July, (*d*) infectious female mosquito population based on different patterns of precipitation in September, (*e*) infectious bird population based on different patterns of precipitation in July and (*f*) infectious bird population based on different patterns of precipitation in September.

Taking the daily temperature and precipitation into consideration, the intraspecific competition will control the development of the vector and consequently hinder the transmission of WNV to some extent. In figure 4*b*, a strong intraspecific competition will shrink the peak of infectious mosquitoes from around 23 to 3. A larger *ρ* has a slight influence to decrease the vector abundance and the spread of WNV, and this influence exhibits only at the peaks of total mosquitoes, infectious birds and mosquitoes (figure 5). Figure 6 indicates that an excess of precipitation can actually impose restrictions on vector production and WNV spread in the population.

## 4. Discussion

This research has demonstrated that temperature, precipitation and intraspecific competition among pre-adult mosquitoes in SWMP are key factors in predicting the WNV vector abundance and the occurrence of WNV in the bird population. Then, for these factors, proactive measures can be taken to control mosquitoes and the spread of WNV. The measures for increasing the intraspecific competition of larvae in SWMP include clearing stagnant water in shallow regions of SWMP, preventing excess nutrients and pollutants from entering the pond, using mechanical aerators to generate water movement, introducing top feeding fish or other predators [51]. Also, based on weather forecasts and our weather-driven model, the prediction of vector abundance and WNV activity will be useful for public health agencies to make a decision on the prevention and control of WNV, such as the use of larvicides and pesticides and encouraging individuals to take personal protection measures including wearing long sleeves and using an insect repellent containing DEET. Certainly, regular monitoring of the vector population in SWMP from May to September is critical to guiding the choice of these prevention and control measures.

In model (2.2), when the intraspecific competition is weak, with the rate less than κ*, virus introduction can lead to an outbreak of WNV and actions to control the spread of the disease would be needed. When a rate greater than κ* occurs, the abundances of larvae and infected mosquitoes decrease due to fierce intraspecific competition, and there are not adequate mosquitoes available to act as a vector of WNV to spread the disease. In model (2.3.4), the intraspecific competition varies with respect to the time-dependent precipitation. In such a situation, the intraspecific competition can be stronger or weaker at different times, and one cannot simply conclude that vector populations and the WNV transmission will be persistently controlled because the effects of the intraspecific competition also will change over time.

Temperature and precipitation combined are complex. Figure 3*a* depicts how weather conditions influence the occurrence of a WNV outbreak when considering a specific SWMP ($\bar{\kappa }$ and *ρ* are constants). With suitable temperature (20–30^°^*C*) an outbreak of WNV will occur, regardless of precipitation, whereas if the temperature is lower or higher than the suitable range, moderate precipitation (0–30 mm) will enhance the potential occurrence of a WNV outbreak. Under the same weather conditions, habitats for SWMP can differ for egg deposition and larval development, leading to different basic reproduction numbers (figure 3*b*). The intraspecific competition rate $\bar{\kappa }$ plays a principal role on ${\hat{R}}_{0}$. A larger $\bar{\kappa }$, such as a deeper pond having more adequate surface water movement, will suppress the reproduction of the mosquitoes and accordingly prevent a WNV outbreak. The effects of precipitation on SWMP and the spread of WNV are of great importance. Under moderate temperature, the interaction effects of precipitation and SWMP are depicted in figure 3*c*,*d*. Moderate precipitation (0–30 mm) will provide more standing water to promote the transmission of WNV; otherwise too much precipitation, for a pond which is sensitive to precipitation, will dilute or eliminate standing water and result in the prevention of a disease outbreak.

Moderate precipitation promotes the spread of the virus and increases vector abundance (figure 6); nevertheless, excess precipitation plays a role in controlling the transmission of WNV and vector abundance. The impact of precipitation is more remarkable in July; with heavier precipitation (60 mm or more daily), the total number of female mosquitoes will decrease rather than rise. A possible explanation is that temperature in July is more suitable for mosquito development and the spread of disease. In September, the weather cools and does not support mosquito survival. When the temperature is suitable for the development of mosquitoes, heavy precipitation suppresses the mosquito population, while moderate precipitation and suitable temperature indicate a need to monitor mosquito abundance and reduce mosquito populations through larviciding. If temperature is low, precipitation may have little impact on mosquito development.

Frequent monitoring and surveillance activities are needed when the temperature is warm, higher than 20^°^*C*. Under such temperature modes, if accompanied by moderate precipitation, it is more likely to trigger the reproduction of WNV vectors and the spread of the virus in the following few days. In this situation, some control actions such as larviciding may be taken to control mosquito abundance in a timely manner. However, if precipitation is quite heavy, there may not be a need to take action to reduce larvae because heavy precipitation will dilute standing water and reduce habitats for larvae. It is worth noting that following such weather patterns, monitoring WNV larvae in SWMP is still needed due to moderate temperatures, but concentrated efforts on larviciding is probably unnecessary. Additionally, applications of aeration and larvicide in the middle of the season may be more effective than applications in the spring or fall, as the weather at this time is suitable for the development of WNV vectors.

**Figure 7.**
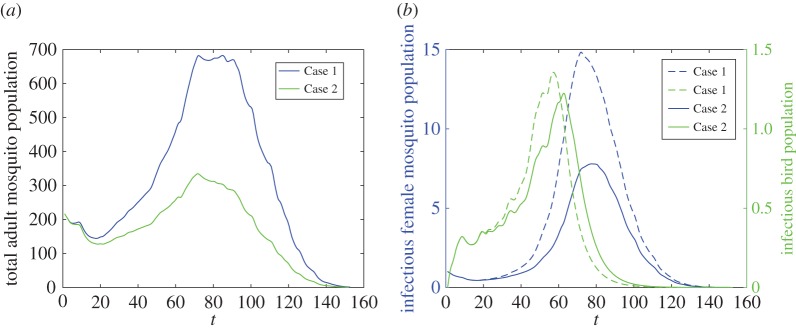
Mosquito abundance and the transmission of WNV in a region with three SWMP. Case 1: SWMP 1: ${\bar{\kappa }}_{1}=0.00004$, SWMP 2: ${\bar{\kappa }}_{2}=0.0005$ and SWMP 3: ${\bar{\kappa }}_{3}=0.002$; Case 2: SWMP 1: ${\bar{\kappa }}_{1}=0.0002$, SWMP 2: ${\bar{\kappa }}_{2}=0.0005$ and SWMP 3: ${\bar{\kappa }}_{3}=0.002$. (*a*) Total adult mosquitoes and (*b*) infectious female mosquitoes and birds.

Usually, a region has multiple SWMP and the characteristics of these ponds also play a role in the transmission of WNV. Each SWMP has an intraspecific competition rate and the harmonic mean (HM) of all these competition rates serves as a representative of all SWMP in the whole region. With the same effect of intraspecific competition rate, a larger HM contributes to decreasing larval populations and infectious mosquitoes and birds. Moreover, to increase the HM, the most effective way is to take actions on the pond in which the intraspecific competition rate is smallest. For instance, in a region with several SWMP, the size of stagnant surface water, the concentration of organics, the population of predators and existence of vegetation will differ in each pond. An economical and effective strategy to control larvae is targeting the SWMP with the weakest intraspecific competition, i.e. the pond holding more standing water with plenty of organics, free of predators and within areas of some vegetation. Applying proactive measures, such as adding top feeding fish and clearing the water body will alleviate the overall severity of WNV and mosquito population. Figure 7 shows that for a region possessing three SWMP with different intraspecific competition rates, only increasing the smallest ${\bar{\kappa }}_{1}$ of SWMP 1 from 0.00004 to 0.0002 could reduce the number of total mosquitoes and infectious vectors and hosts for the overall region.

This work provides insight into how to predict and control mosquito abundance and the transmission of WNV based on temperature, precipitation and SWMP in a region. Measures to promote intraspecific competition among pre-adult mosquitoes, such as refreshing the shallow regions of water in a pond and using aeration to create wave action or water movement, can be taken to reduce the mosquito population and the spread of WNV. There are other factors we have not considered in this work. Wind patterns, the dispersion of birds, elevation variation in birds and mosquito population, all remain to be investigated in future work. The model output is not validated due to the lack of available infection data for birds and mosquitoes, and additional research needs to be conducted in future to overcome this issue.

## 5. Conclusion

Moderate temperature and precipitation will increase the potential of the basic reproduction number being greater than one, increasing the mosquito population and consequently the potential for an outbreak of WNV. On the contrary, excess precipitation could reduce mosquito population, which may lead to a lower peak value of infectious mosquitoes and birds. A smaller intraspecific competition rate (an indicator of the SWMP properties) leads to a larger mosquito population and more infectious birds and mosquitoes. This work can be used to guide WNV programmes in local health units where monitoring of standing water and larviciding are often used to control mosquito populations and the spread of WNV. In addition to temperature, precipitation and SWMP, the influence of other factors such as wind patterns and elevation on the abundance of mosquitoes and the transmission of WNV are worth taking into account in future work. Also, demographics of birds including migrations and the long-term effects of these influential factors will be considered in future work.

## Figures and Tables

**Figure 8.**
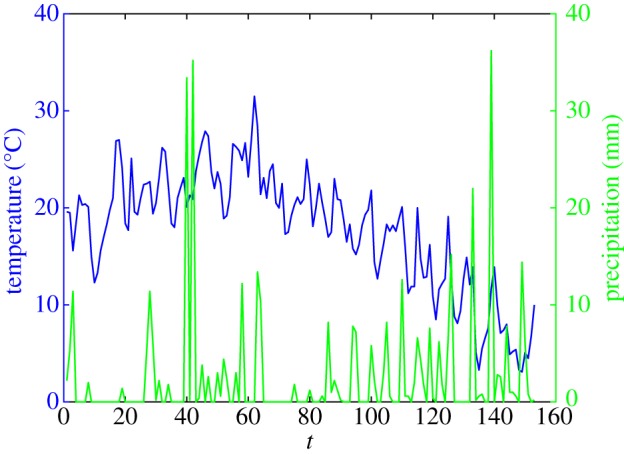
Weather variations from June to October in 2006 in the GTA.

## Data accessibility

The datasets supporting this article are deposited at Dryad Digital Repository: http://dx.doi.org/10.5061/dryad.9nb56 [52].

## Appendix A. Disease-free equilibrium and *R*_0_ in mosquito–bird model (2.2) without weather factors

For the model (2.2), if *r*_m_*δ*/2*d*_m_−(*d*_l_+*δ*)<0, the model has a unique equilibrium point *E*_0_=(0,0,0,0,0,*S*_*b*_0__,0,0,0), where *S*_*b*_0__ is any given initial density of birds. The *E*_0_ has eigenvalues 0 (multiplicity 3), −*d*_m_, $-{\tilde{d}}_{m}$, −(*d*_m_+*k*), −(*μ*+*γ*) and the roots of the equation:

A 1 $$2\lambda^{2}+2(d_{l}+d_{m}+\delta )\lambda +2d_{l}d_{m}+2d_{m}\delta -r_{m}\delta =0.$$

All parameters are positive in a biological sense, all the roots of (A 1) have negative real parts, and DFE *E*_0_ is locally stable.

If *r*_m_*δ*/2*d*_m_−(*d*_l_+*δ*)>0, that is in addition to death and maturation working on reducing the rate of *L*_m_, intraspecific competition is also involved in playing a part, then the model (2.2) has two DFE points, *E*_0_ and *E*_1_=(*L*_*m*_0__,*M*_*m*_0__,*S*_*m*_0__,0,0,*S*_*b*_0__,0,0,0), where *L*_*m*_0__=*r*_m_*δ*/2*d*_m_−(*d*_l_+*δ*)/*κ*, $M_{m_{0}}=\delta [r_{m}\delta /2d_{m}-(d_{l}+\delta )]2{\tilde{d}}_{m}\kappa$, *S*_*m*_0__=*δ*[*r*_m_*δ*/2*d*_m_−(*d*_l_+*δ*)]2*d*_m_*κ* and *S*_*b*_0__ is any given initial density of birds. Here, *E*_0_ has a positive real part eigenvalue due to (A 1), thus *E*_0_ is unstable. The local stability of *E*_1_ is determined by the basic reproduction number *R*_0_ which can be obtained from the next-generation matrix for system (2.2).

Using the notation of van den Driessche & Watmough [50], with the infected variables (*E*_m_,*I*_m_,*I*_b_) in model (2.2), F denotes the rate of new infections and V denotes the rate of transfer between compartments:

$$F=\left[\begin{array}{c} b_{m}\beta_{m}S_{m}\frac{I_{b}}{N_{b}+A}\\ 0\\ b_{m}\beta_{b}I_{m}\frac{S_{b}}{N_{b}+A} \end{array}\right]\;\mathrm{and}\;V=\left[\begin{array}{c} kE_{m}+d_{m}E_{m}\\ -kE_{m}+d_{m}I_{m}\\ \mu I_{b}+\gamma I_{b} \end{array}\right],$$

The corresponding linearized matrices at the DFE *E*_1_ are

$$F=\left[\begin{array}{ccc} 0 & 0 & b_{m}\beta_{m}\frac{S_{m_{0}}}{N_{b_{0}}+A}\\ 0 & 0 & 0\\ 0 & b_{m}\beta_{b}\frac{S_{b_{0}}}{N_{b_{0}}+A} & 0 \end{array}\right]\;\mathrm{and}\;V=\left[\begin{array}{ccc} k+d_{m} & 0 & 0\\ -k & d_{m} & 0\\ 0 & 0 & \mu +\gamma \end{array}\right].$$

Then, the basic reproduction number *R*_0_ is defined as the spectral radius of the matrix *FV* ^−1^,

A 2 $$R_{0}=\sqrt{\frac{b_{m}\beta_{m}(S_{m_{0}}/(N_{b_{0}}+A))(k/(k+d_{m}))}{\left(\mu +\gamma \right)}\frac{b_{m}\beta_{b}(S_{b_{0}}/(N_{b_{0}}+A))}{d_{m}}},$$

where *S*_*m*_0__=*δ*[*r*_m_*δ*/2*d*_m_−(*d*_l_+*δ*)]2*d*_m_*κ* and *S*_*b*_0__ is any given initial density of birds.

## Appendix B. ${\hat{R}}_{0}$ in mosquito–bird model with temperature and precipitation

For fixed temperature and precipitation, the basic reproduction number is

A 3 $${\hat{R}}_{0}=\sqrt{\frac{k\beta_{m}\beta_{b}S_{b_{0}}b_{m}{\left(T\right)}^{2}\delta (T)[r_{m}(T)\delta (T)/2d_{m}(T)-(d_{l}(T)+\delta (T))]}{2{\left(N_{b_{0}}+A\right)}^{2}(\mu +\gamma )d_{m}{\left(T\right)}^{2}(k+d_{m}(T))\kappa (P)}};$$

the threshold ${\hat{R}}_{0}=1$ still plays a significant role in an occurrence of a WNV outbreak.

First, we explore the variation of ${\hat{R}}_{0}$ along with precipitation:

A 4 $$\frac{\partial {\hat{R}}_{0}}{\partial P}=\frac{\partial {\hat{R}}_{0}}{\partial \kappa }\frac{\partial \kappa }{\partial P}=\frac{\partial {\hat{R}}_{0}}{\partial \kappa }\frac{2\kappa^{2}\rho (P-P_{l})\exp[-{\left((P-P_{l})/{\mathrm{Var}}_{Pl}\right)}^{2}]}{{\mathrm{Var}}_{Pl}^{2}(1+\rho )\bar{\kappa }}.$$

Apparently, $\partial {\hat{R}}_{0}/\partial \kappa <0$; accordingly, $\partial {\hat{R}}_{0}/\partial P>0$ when *P*<*P*_l_ and $\partial {\hat{R}}_{0}/\partial P<0$ when *P*>*P*_l_, and $\lim_{P\to \infty }(\partial {\hat{R}}_{0}/\partial P)=0$. That means ${\hat{R}}_{0}$ increases as precipitation *P* increases starting from 0; once crossing the optimal value *P*_l_, ${\hat{R}}_{0}$ begins to decrease and gradually approaches a constant.

Then for SWMP, both $\bar{\kappa }$ and *ρ* are indicators to reflect the properties of a pond. We look into how each of them act in deciding the value of ${\hat{R}}_{0}$. Similarly,

$$\begin{array}{rl} \frac{\partial {\hat{R}}_{0}}{\partial \bar{\kappa }} & =\frac{\partial {\hat{R}}_{0}}{\partial \kappa }\frac{1+\rho }{1+\rho \exp[-{\left((P-P_{l})/{\mathrm{Var}}_{Pl}\right)}^{2}]}<0,\\ \frac{\partial {\hat{R}}_{0}}{\partial \rho } & =\frac{\partial {\hat{R}}_{0}}{\partial \kappa }\frac{\bar{\kappa }(1-\exp[-{\left((P-P_{l})/{\mathrm{Var}}_{Pl}\right)}^{2}])}{{{\left(1+\rho \exp[-((P-P_{l})/{\mathrm{Var}}_{Pl}\right)}^{2}])}^{2}}\leq 0 \end{array}$$

and $\lim_{\rho \to \infty }(\partial {\hat{R}}_{0}/\partial \rho )=0$. Note that ${\hat{R}}_{0}$ decreases as $\bar{\kappa }$ or *ρ* increases, and it will approach a constant when *ρ* is sufficiently large; a special case is that ${\hat{R}}_{0}$ is always constant with *P*=*P*_l_, no matter how *ρ* changes.

## Appendix C. The daily mean temperature and daily total precipitation from June to October in 2006 in the Greater Toronto Area

See figure 8.
